# Navigation-Assisted and Robotic Total Hip, Knee, and Unicompartmental Arthroplasty: Reconciling Positioning Accuracy with Biomaterial Performance and Long-Term Implant Survival

**DOI:** 10.3390/bioengineering13101182

**Published:** 2026-10-10

**Authors:** Yasaman Tavakoli, Fatemeh Kanaani Nejad, Kartik Varsani, Roshan Latifi, Mohammad Poursalehian, Jad Ismail, Robert A. Sershon

**Affiliations:** 1 Department of Medicine, Mazandaran University of Medical Sciences, Sari 48157-33971, Iran; y.tavakoli@mazums.ac.ir; 2 Medical Imaging Research Center, Shiraz University of Medical Sciences, Shiraz 71348-14336, Iran; fkanaani@sums.ac.ir; 3 Philadelphia College of Osteopathic Medicine, 4190 City Avenue, Philadelphia, PA 19131, USA; kartikvarsani@gmail.com; 4 Department of Orthopaedic Surgery, Aalborg University Hospital, 9000 Aalborg, Denmark; roshan.latifi@me.com; 5 Anderson Orthopaedic Research Institute, Alexandria, VA 22306, USA; jismail@aori.org (J.I.); rsershon@andersonclinic.com (R.A.S.)

**Keywords:** robotic arthroplasty, surgical navigation, implant positioning, polyethylene wear, edge loading, tribocorrosion, hydroxyapatite coating, cementless fixation, radiostereometric analysis, implant survivorship

## Abstract

Navigation-assisted and robotic systems improve component positioning in total hip (THA), total knee (TKA), and unicompartmental knee arthroplasty (UKA), but whether this improves biomaterial performance and implant survival is unclear. This structured narrative review asks for which bearing materials and fixation interfaces positioning accuracy plausibly alters the degradation and fixation pathways that govern survival, and how strong the evidence is for each link. We propose a conceptual model linking positioning to the local mechanical environment, material-specific degradation, biological response, and survival, and grade each link’s evidence by study design. Enabling technologies consistently reduce outliers. Position-dependent degradation is well documented for conventional polyethylene, ceramic-on-ceramic, and metal-on-metal bearings, mainly from simulator, retrieval, and cohort studies. Highly cross-linked polyethylene appears to attenuate the wear penalty of malposition, although rim fatigue and impingement remain position-sensitive and vitamin E stabilization has no proven additional clinical benefit. In cementless TKA, varus alignment is associated with greater early tibial migration, a validated surrogate for loosening. Clinical translation remains limited: trials and registries show no consistent reduction in all-cause revision after TKA or THA, with lower dislocation after THA and a more favorable UKA signal as exceptions. Precision may matter most for position-sensitive bearings and cementless interfaces.

## 1. Introduction

Navigation-assisted and robotic systems are increasingly used in total hip arthroplasty (THA), total knee arthroplasty (TKA), and unicompartmental knee arthroplasty (UKA) to reduce variability in component positioning and to enable patient-specific planning. Their radiographic advantages are consistently reported [1,2,3], but whether greater accuracy improves bearing performance, fixation, patient-reported outcomes, and long-term implant survival remains uncertain.

Existing systematic reviews and meta-analyses compare robotic or navigated with conventional surgery in terms of alignment, function, complications, and revision [2,3,4,5], and broader reviews, including one in this journal, describe robotics and biomaterials as parallel technological advances [6,7]. To our knowledge, previous reviews have not systematically integrated implant material and fixation interface as potential effect modifiers linking positioning accuracy with material degradation and long-term implant survival. This gap matters because the classic evidence linking malposition to wear and loosening was generated largely with conventional polyethylene, cemented fixation, and earlier bearing couples. Highly cross-linked and vitamin E-stabilized polyethylene, contemporary ceramics, porous and additively manufactured surfaces, and hydroxyapatite (HA) coatings may each respond differently to the same positioning error.

The central question of this review is therefore: for which bearing materials and fixation interfaces does improved positioning plausibly change the mechanical loading, degradation, and fixation pathways that determine long-term survival, and what type and strength of evidence supports each link? We first describe the review methodology and an explicit evidence hierarchy (Section 2), then propose a conceptual model (Section 3). We summarize the positioning benefit (Section 4); analyze position-dependent degradation material by material, with attention given to how wear is measured (Section 5); examine fixation interfaces (Section 6); and assess clinical translation and its detection (Section 7, Section 8, Section 9 and Section 10).

## 2. Methods

### 2.1. Literature Search

This review was designed as a structured narrative review meeting the criteria of the Scale for the Assessment of Narrative Review Articles (SANRA) [8]. PubMed/MEDLINE was searched from inception to 28 September 2026 with three combined searches (Appendix A, Table A1): enabling technology AND procedure AND outcome (4151 records); procedure AND malposition AND degradation or fixation outcome (1222 records); and procedure AND bearing or fixation material AND study design (1488 records). The second and third searches did not require an enabling-technology term, because most material-level evidence on wear, corrosion, and fixation is independent of enabling technology. Reference lists of included reviews and key trials were hand-searched, and targeted searches were performed for registry reports and guidelines.

### 2.2. Eligibility and Study Selection

We included English-language, peer-reviewed studies of adult primary THA, TKA, or UKA that reported (a) positioning accuracy with navigation, robotics, or patient-specific instrumentation; (b) the relationship between component position and wear, degradation, fixation, or migration; (c) clinical, registry, patient-reported, or economic outcomes of enabling technology; or (d) material or interface behavior relevant to (b) from simulator, finite-element, cadaveric, animal, or retrieval studies. For comparative clinical questions, randomized controlled trials (RCTs), meta-analyses of RCTs, and national registries were prioritized; smaller series were included when they provided the only data on a mechanism or subgroup. Revision arthroplasty, non-arthroplasty applications, conference abstracts, and case reports were excluded, except retrieval series documenting a failure mechanism; hip resurfacing studies were included only as evidence of metal-on-metal degradation mechanisms. Consistent with the narrative design, records were not screened exhaustively or in duplicate. Studies were selected for relevance to the review question, with priority given to higher evidence tiers (Section 2.3), and the documented searches were used to check coverage and to identify additional eligible studies.

### 2.3. Evidence Classification

Because the question spans bench and clinical science, each cited primary study was assigned to one of four evidence tiers adapted from established hierarchies (Table 1) [9]; reviews, economic analyses, and guidelines were not tiered. Throughout the review, the tier is stated where a conclusion rests mainly on Tier III or IV evidence. Radiographic alignment, migration, and simulator wear are treated as surrogate endpoints that generate hypotheses about clinical outcomes rather than substitute for them, unless they have been validated against revision [10,11,12]. No quantitative pooling or formal risk-of-bias assessment was performed because designs, endpoints, and test conditions were too heterogeneous; this limitation is discussed in Section 12.

## 3. Conceptual Framework: From Positioning to Survival

**Figure 1:**
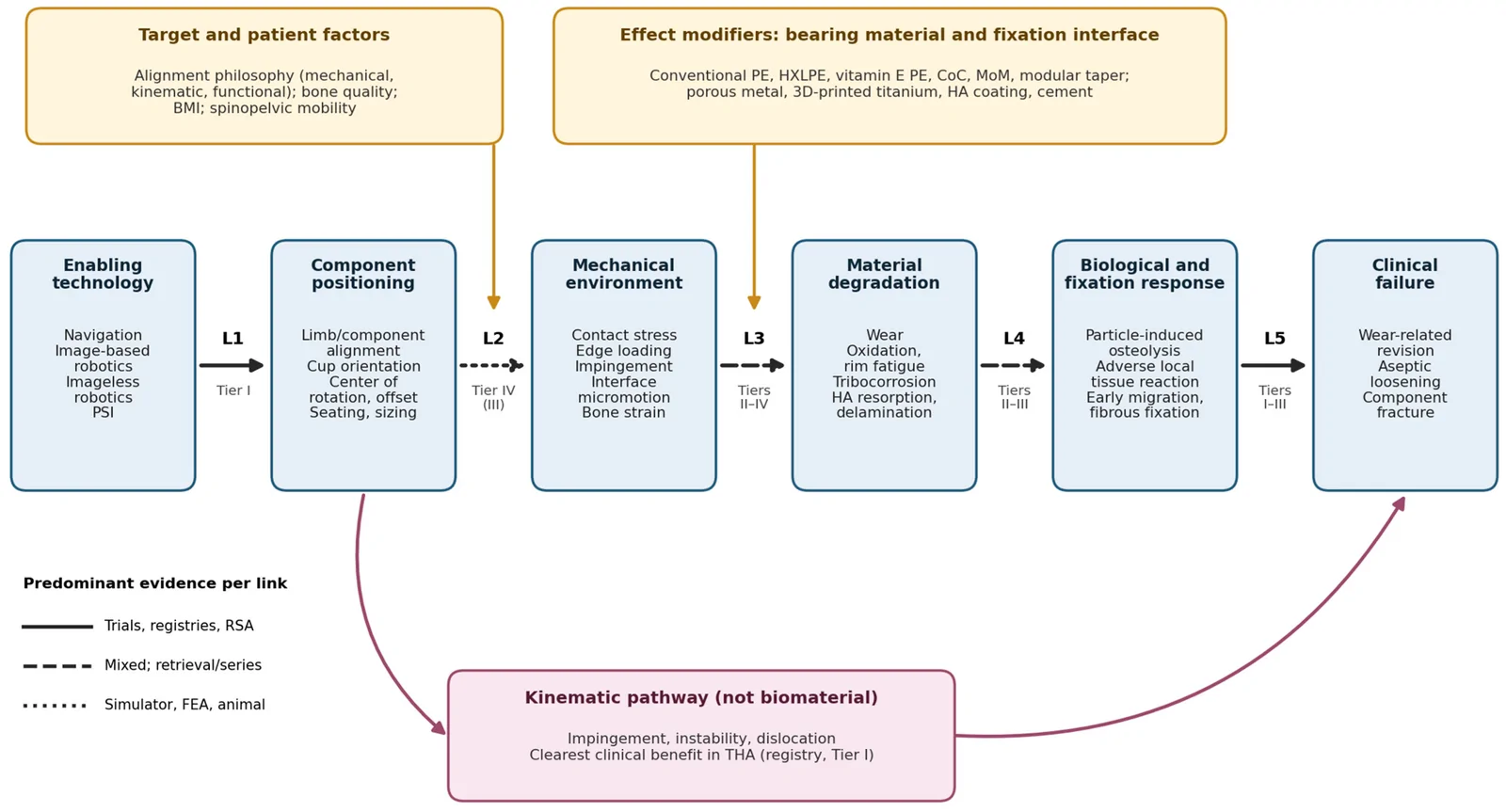
Conceptual model linking enabling technology, component positioning, the local mechanical environment, material-specific degradation, biological response, and implant failure. Links L1–L5 are drawn by the predominant evidence supporting each: solid, Tier I–II (trials, registries, RSA); dashed, mixed evidence anchored in Tier III retrieval studies and series; dotted, Tier IV (simulator, finite-element, cadaveric, animal). Bearing material and fixation interface modify Link 3; alignment target and patient factors modify Link 2. The kinematic pathway to instability bypasses material degradation. BMI, body mass index; CoC, ceramic-on-ceramic; FEA, finite-element analysis; HA, hydroxyapatite; HXLPE, highly cross-linked polyethylene; MoM, metal-on-metal; PE, polyethylene; PSI, patient-specific instrumentation; RSA, radiostereometric analysis.

Figure 1 summarizes the pathway through which enabling technology could influence implant survival. Link 1 connects technology to positioning accuracy and is supported by Tier I evidence (Section 4). Link 2 connects positioning to the local mechanical environment: contact stress and edge loading at the articulation, and micromotion, strain, and load sharing at the bone–implant interface. This link rests mainly on Tier IV evidence with Tier III support. Link 3 connects the mechanical environment to material-specific degradation, namely wear, oxidation and fatigue, tribocorrosion, and coating degradation; it is where material and interface properties act as effect modifiers (Tiers II–IV). Link 4 connects degradation and interface loading to the biological and fixation response, including particle-induced osteolysis, adverse local tissue reactions, early migration, and fibrous rather than osseous fixation (Tiers II–III). Link 5 connects these processes to clinical failure through aseptic loosening, wear-related revision, and instability (Tiers I–III).

Two implications follow. First, a positioning benefit can reach survival only if every link holds for a given material–interface combination, so the chain is only as strong as its weakest, material-specific link. Second, the same malposition can have different consequences depending on the material (Table 2): position sensitivity is high for conventional polyethylene, ceramic-on-ceramic (CoC), and metal-on-metal (MoM) bearings, and cementless interfaces, but attenuated for the wear of highly cross-linked polyethylene (HXLPE). Dislocation, the clearest clinical benefit in THA, bypasses Links 3 and 4; it is a kinematic rather than a biomaterial endpoint (Section 7.2).

## 4. Positioning Accuracy: The Established Benefit

In TKA, robotic assistance reduces mechanical-axis outliers (risk ratio [RR] 0.43; 95% confidence interval [CI] 0.27–0.67 across 12 RCTs) [2]. The effect is platform-dependent; “robotic” should not be treated as a single category [3]. Image-based and imageless systems achieve comparable accuracy, with most bone cuts within 2 mm or 2° of plan [52,53,54], and comparative, randomized, and cadaveric studies confirming reduced deviation from plan [1,55,56,57]. Computer navigation reduces hip–knee–ankle outliers to a similar degree (RR 0.45 versus standard guides across 90 RCTs), whereas patient-specific instrumentation performs less consistently [58,59,60]. In THA, an RCT found that robotic assistance reduced root-mean-square error for cup anteversion (2.6° versus 8.9°) and inclination (1.3° versus 5.3°) and improved restoration of center of rotation and offset [61]. Robotic guidance also outperformed navigation and manual technique for functional safe-zone targeting [62].

Robotic platforms also change how the soft-tissue envelope and polyethylene insert are managed. Ligament release rates fall (7% versus 50% in one RCT), thinner inserts are used [63,64], and cadaveric sensor testing shows more balanced mediolateral loading [65]. Whether this balance matters for the bearing is untested, and sensor-guided balancing has not improved one-year function in two RCTs [66,67]. Thinner inserts preserve bone but also reduce the polyethylene thickness available to resist creep and fatigue—a trade-off that has not been examined in robotic cohorts.

## 5. Bearing Surfaces: Position-Dependent Degradation by Material

### 5.1. How Wear Is Measured: Simulator, Retrieval, and Clinical Evidence Are Not Interchangeable

Wear values in this literature come from sources that are not directly comparable. Hip simulators run to International Organization for Standardization (ISO) standard 14242-1 reproduce idealized gait with the head centered in the cup. Under these conditions, alumina CoC bearings wear approximately 0.1 mm^3^ per million cycles, with none of the damage seen on retrieved bearings; adding swing-phase microseparation produced 1.24–1.74 mm^3^ per million cycles together with the stripe wear and bimodal debris observed clinically [27]. Increasing cup inclination alone often fails to reproduce the wear increases found in retrievals. In MoM bearings, the effect of inclination depended on head size and was outweighed by translational malpositioning [68]. In another study, neither CoC nor conventional or vitamin E-stabilized polyethylene e wore more at steep inclination [23]. Protocols that combine rotational and translational malposition are therefore needed to test position sensitivity [69]. Gravimetric wear rate can also miss the relevant failure mode: under edge loading, an aged conventional liner developed rim damage and cracking despite a lower gravimetric wear rate than under standard loading [70].

The same caution applies to the knee. Force- and displacement-controlled versions of ISO 14243 produce different kinematics, and predicted wear under force control was more than double that under displacement control, so values from different standards should not be compared [71]. The ISO input profiles differ from in vivo TKA gait [72], and simulator wear scars resembled only about 11% of retrieved inserts [73]. Simulators and retrievals also disagree on alignment. Varus malalignment of 4° produced similar or lower simulator wear than neutral alignment [74,75], whereas retrieved tibial components implanted in more than 3° of varus showed almost twice the volumetric penetration rate [14]. Rotational mismatch and a 10° posterior slope did increase simulator wear [74], consistent with retrieval data on slope and joint-line elevation [76]. Finite-element models add estimates of contact stress, for example a 145.9% increase in peak stress with 5° of varus tilt [15], but they predict stress rather than wear.

Clinical measurements of head penetration by radiography or radiostereometric analysis (RSA) capture real kinematics but are confounded by activity, bedding-in, and creep, and they are rarely stratified by the technology used for implantations. In the sections that follow, simulator findings are therefore treated as evidence of mechanism, retrieval findings as evidence that a mechanism operates in vivo, and clinical wear or revision data as evidence of clinical relevance.

### 5.2. Conventional Polyethylene: The Origin of the Positioning Rationale

The wear–alignment relationship was established for conventional ultra-high-molecular-weight polyethylene. In THA, mean linear wear rises by about 40% when cup abduction exceeds 45°, and a prospective series reported 0.12 versus 0.18 mm/year below and above 45° [13,77]. Steeply inclined cups have been associated with more osteolysis, asymmetric wear, and migration [78], and a cohort study linked abduction to accelerated wear and severe acetabular bone defects [79]. In TKA, tibial varus of more than 3° and increased posterior slope and joint-line elevation are associated with greater polyethylene wear in retrieved inserts [14,76]. The biological consequence is dose-dependent. Submicron particles activate macrophage nuclear factor-κB (NF-κB) and NLRP3 inflammasome signaling and osteoclastogenesis [80,81], osteolysis is uncommon below approximately 0.1 mm/year of linear wear [82,83], and aseptic loosening remains a leading cause of late THA revision [84]. These Tier II–III data consistently associate malposition with accelerated wear of conventional polyethylene to a clinically relevant degree. They do not establish that robotic accuracy reduces wear-related revision with contemporary materials.

### 5.3. Highly Cross-Linked and Vitamin E-Stabilized Polyethylene: Attenuated, Not Eliminated, Position Sensitivity

Cross-linking changes this relationship. Compared with conventional polyethylene, HXLPE produced linear wear of 0.03 versus 0.17 mm/year and wear-related reoperation of 0% versus 12% at 15 years in an RCT [21], and wear of 0.034 versus 0.181 mm/year at a minimum of 20 years in a double-blind RCT [85]. In the Australian registry, the 16-year cumulative revision rate was 6.2% versus 11.7% [22]; earlier 10-year trial data are consistent [86,87]. Radiographic and RSA studies found no significant association between cup orientation and HXLPE wear at 10 to 12 years (Tier II) [16,17]. In a simulator, HXLPE wear did not increase at 75° of inclination, even though finite-element analysis predicted higher liner stress (Tier IV) [88].

Four residual mechanisms are position-relevant and temper this conclusion. The first is fatigue and fracture. Cross-linking reduces ductility and resistance to fatigue crack initiation and propagation, and annealing may be somewhat less detrimental than remelting (Tier IV) [20]. Retrieved HXLPE liners have cracked or fractured at thin, unsupported rims, with vertical cup orientation and rim loading implicated [18,19]. These failures are documented mainly in retrieval case series, and gravimetric wear measurements can miss rim cracking, as simulator testing of conventional liners has shown [70]. The second is oxidation. First-generation annealed liners oxidize in vivo, particularly at the rim, and rim oxidation increases with implantation time and accompanies fatigue damage [89,90]. However, retrieved annealed and remelted liners implanted for more than 10 years show similar penetration despite higher oxidation of the annealed liners, so the clinical relevance of oxidation is uncertain [91]. The third is impingement. Neck–liner impingement is present on more than half of retrieved acetabular components and on 94% of those revised for dislocation, and it is associated with cup anteversion and head–neck ratio [92,93]. Impingement damage depends on position and geometry rather than on wear resistance. The fourth is head size and liner thickness. Larger heads on HXLPE produced greater volumetric wear at 10 to 14 years [94], although thin vitamin E-stabilized liners with 36 to 44 mm heads showed no increase in penetration at 5 years [95]. Orientation-dependent wear has also been detected with modern liners at 5 to 16 years, for example, with anteversion of 25° or more [24,96,97].

The clinical support for attenuation is weaker in the knee. In the Australian registry, cross-linked TKA inserts had lower 10-year revision rates only for some designs [98], and a large US registry and a meta-analysis found no difference in revision [99,100].

Vitamin E stabilization was introduced to improve oxidative stability without further loss of mechanical properties. In a simulator, wear of vitamin E-stabilized HXLPE was essentially unchanged between 45° and 80° of inclination [23]. In one RCT, head penetration did not differ significantly from standard HXLPE at five years [25] but was lower at 10 years [101]. Low penetration has been reported at 10 years or more in a multicenter cohort [102] and at 14 years in an RSA trial without a non-vitamin E comparator [103]. Registry survival was similar to that of moderately cross-linked polyethylene at seven years [26], although national registry data indicate that irradiation dose and stabilization method influence aseptic revision risk [104]. The simulator result suggesting position insensitivity should be read with caution, because conventional polyethylene also did not wear more at steep inclination in the same test [23]; this suggests that the test (steep inclination without microseparation) may have lacked sensitivity to position, rather than demonstrating a property unique to vitamin E-stabilized material. Overall, the evidence supports attenuation of the wear penalty of modest malposition with HXLPE (Tiers I–II), a plausible but clinically unconfirmed further advantage for vitamin E stabilization (Tiers II–IV), and persistence of position-dependent rim fatigue and impingement (Tiers III–IV).

### 5.4. Ceramic-on-Ceramic Bearings: Edge Loading, Stripe Wear, and Fracture

CoC bearings have the lowest wear under standard conditions but are intolerant of edge loading. In simulators, microseparation increases the wear of third-generation alumina by more than an order of magnitude [27], whereas wear of 36 mm BIOLOX delta bearings rose from below 0.1 to about 0.22 mm^3^ per million cycles, remaining low [105]. Finite-element models show that edge loading begins once medial–lateral separation exceeds about 30 µm, with contact pressure rising from 66 MPa toward 205 MPa and worsening with inclination [106], and simulator studies show that translational and rotational malposition interact (Tier IV) [69]. Retrievals confirm that stripe wear arises from edge loading in vivo [107]. In 54 retrieved bearings, posterior edge loading predominated, and anteversion was a more important determinant of wear than inclination (Tier III) [28], consistent with a computational “landing zone” that is smaller than the Lewinnek zone and more sensitive to anteversion [108]. Squeaking was associated with cups outside a target zone in one case–control study [29] but not in another that used computed tomography (CT) [30], and fractures of polyethylene–ceramic composite (sandwich) liners have followed neck–rim impingement [109]. Position is therefore a contributing, but not the sole, determinant of ceramic-specific failure. Because the translational component of edge loading, governed by offset, soft-tissue tension, and center of rotation, matters as much as cup angle, CoC bearings are a plausible setting in which robotic restoration of center of rotation and offset [61] could affect bearing performance. No study has tested this directly.

### 5.5. Metal Surfaces and Tribocorrosion

MoM bearings provide the clearest clinical demonstration that position alters material degradation. Inclination above 55° and a small arc of cover were associated with higher serum cobalt and chromium [31], and insufficient version also raised ion levels [32]. Early failure from adverse reactions to metal debris was associated with small components, high anteversion, high ion levels, and excess wear (Tiers II–III) [110], while simulator wear increased ninefold at 60° of inclination and 17-fold with added microseparation (Tier IV) [33]. This precedent shows that the positioning–degradation–biological response chain can close clinically when a material is intolerant of edge loading.

The situation differs at the modular head–neck taper of contemporary metal-on-polyethylene THA. Taper damage results from mechanically assisted crevice corrosion, and in retrieval series its predictors are implantation time, neck flexural rigidity, and alloy combination [34]. It can cause adverse local tissue reactions [111], and registry data associate metal and larger femoral heads with higher revision rates for adverse reactions to metal debris [112]. However, a cohort with magnetic resonance imaging surveillance found no association between cup inclination and taper-related tissue reactions [35]. We found no evidence that component positioning determines taper tribocorrosion, and it should not be assumed to be a pathway through which robotic accuracy improves survival.

## 6. Fixation Interfaces: Where Precision May Matter Most

### 6.1. Primary Stability and the Micromotion Window

Cementless fixation depends on limiting early interface micromotion. Classic animal studies showed bone ingrowth with micromotion of up to about 20–28 µm and fibrous encapsulation at 150 µm or more [113,114]. A systematic review of in vivo data reported mean micromotion of 112 µm in implants that osseointegrated versus 349 µm in those that did not, with wide overlap and no universal threshold [115]; a tolerance of 100–200 µm is commonly assumed in finite-element studies [116]. In models, positioning maps on to this window. In patient-specific finite-element models, 2° of tibial varus increased peak baseplate micromotion by an average of 79% and the bone at risk of failure by 59%, although the ingrowth-compatible area remained larger than that seen in well-fixed retrievals [36]. Resection and tray alignment errors alter predicted micromotion and ingrowth area [117], and larger press-fit and polar gaps increased acetabular micromotion (Tier IV) [118]. In cadaveric femora, undersizing increased stem subsidence by 320% and varus alignment increased cortical strain [119], and robotic femoral preparation improved bone–implant contact (93% versus 60%) [120]. Frictional resistance, often assumed to protect primary stability, depends on surface texture, bone density, and contact stress, with coefficients of 0.37–0.95 reported for additively manufactured titanium [121]. A higher-friction porous coating did not improve cup stability in cadavers [122], so surface engineering cannot fully substitute for accurate seating.

### 6.2. Porous Architecture and Fatigue

Porous surfaces involve a trade-off between biological and mechanical performance. Pore sizes of roughly 200–600 µm and porosity around 60–70% favor ingrowth, but higher porosity lowers implant strength and can reduce osseointegration strength [123,124,125]. Fatigue is the relevant mechanical constraint: additively manufactured Ti-6Al-4V lattices with 68–84% porosity have normalized endurance limits below 0.2, compared with about 0.4 for solid material (Tier IV) [126]. Eccentric or concentrated loading from malposition may therefore consume a larger share of the fatigue margin in highly porous structures, a hypothesis that has not been tested clinically. Porous tantalum (75–80% porosity, with an elastic modulus approaching that of cancellous bone) achieves rapid ingrowth and high early shear strength in animals [127] and durable acetabular fixation clinically [128,129], although a meta-analysis found no consistent biological advantage over porous titanium [130]. Stiffness matching carries its own trade-off, because flexible stems reduce stress shielding but increase proximal interface stress and micromotion [131]. Clinically, a three-dimensional (3D)-printed cementless tibia stabilized on RSA after 2 years and showed high mid- to long-term survivorship in trial and registry data [132,133,134].

### 6.3. Hydroxyapatite Coatings: Coating Quality, Delamination, and Loading

The performance of HA coatings depends on coating quality as much as on the presence of a coating [135]. Thickness governs mechanical integrity. In a canine push-out model, 50 µm coatings had higher shear strength than 200 µm coatings, which failed within the coating and at the coating–metal interface, attributed to residual stress and dissolution (Tier IV) [40]. Crystallinity governs a trade-off between bioactivity and stability. Under 250 µm of controlled micromotion, 50% crystalline HA achieved threefold greater fixation at 16 weeks compared to 75% crystalline HA, a difference that had disappeared by 32 weeks, but it lost more coating (Tier IV) [41]. Delaminated HA can act as a third body: HA granules were embedded in the articulating surface of all six retrieved HA-coated cups in one series, with abrasive wear and osteolysis [42], and delamination has been characterized in a retrieved stem [136].

Clinically, HA coating reduced RSA migration of uncemented tibial components for more than 10 years [43,137] and a calcium phosphate coating reduced femoral radiolucencies in a randomized trial [138]. In contrast, HA-coated cups had a higher risk of revision for aseptic loosening in the Swedish registry (adjusted RR 1.7) [44], HA on uncemented stems did not improve survival in Nordic registry data [139], and the Australian registry effect was design-dependent [140]. Whether malposition-induced shear and micromotion accelerate HA resorption or delamination has not been studied. Because coating loss in the animal model occurred under controlled micromotion [41], this interaction is a plausible and testable reason why coated surfaces might be more position-sensitive than the underlying porous substrate.

### 6.4. Migration as a Validated Surrogate: Linking Alignment to Loosening

RSA migration is the most useful bridge between positioning and late fixation failure because it has been validated against survival. Each additional millimeter of early tibial migration is associated with an 8% increase in revision rate, with thresholds of 0.5 mm (acceptable) and 1.6 mm (unacceptable) at one year [11]; these thresholds were recently validated and refined for cemented and uncemented designs [141]. The corresponding acetabular thresholds are 0.2 and 1.0 mm of proximal migration at two years [12]. Pooled RSA data from 10 RCTs (476 patients) showed that varus-aligned uncemented tibial components migrate more than neutral ones, with divergence increasing over time and uncoated components migrating most [37,38]. This is the strongest evidence that the error robotics reduces is relevant to cementless fixation. The chain has not closed clinically, however. The two randomized RSA comparisons of implantation technique, both in cemented TKA, showed no migration benefit: tibial migration was similar with computer navigation and conventional technique [48], and robotic implantation of a bi-cruciate stabilized design was associated with slightly greater migration at two years (difference 0.21 mm), although migration was acceptable in both groups [49]. Registry analyses found no difference in two-year revision between robotic and non-robotic cementless TKA [39] or in five-year revision for navigated or robotic TKA overall [142]. The only single-design comparison reporting fewer aseptic loosening failures with robotics (0% versus 6.6%) is a single-center retrospective series [143], and a lower septic revision rate after cementless than after cemented robotic TKA [144] is not a biomaterial endpoint. Registry survivorship of cementless and cemented fixation is broadly similar, with design effects outweighing fixation effects [145,146,147].

### 6.5. Cemented Interfaces

For cemented knees, the association between alignment and failure is well documented but mechanistically distinct. Tibial varus of more than 3° was associated with medial bone collapse [45]. Neutral postoperative alignment was associated with lower aseptic failure (0.74% versus 1.7%) [50], and combined tibial and femoral malalignment with failure rates of up to 8.7% versus 0.2% for neutral components (Tier III) [51]. Combined finite-element and cadaveric work suggests that failure after varus tray loading reflects fatigue damage of proximal tibial bone rather than failure of the cement mantle or interface [46]. Conversely, cement–implant debonding, an interface failure, occurred predominantly in well-aligned knees (22 of 25) with one design [47]. Accurate positioning therefore does not protect against interfacial failure driven by cement, implant surface, or technique.

## 7. Clinical Translation: The Accuracy–Outcomes Gap

### 7.1. Patient-Reported Outcomes and Revision

Despite improved accuracy, high-quality clinical evidence has not shown consistent improvement in patient-reported outcome measures (PROMs) or all-cause revision at mid-term follow-up. The multicenter, participant- and assessor-masked RACER-Knee trial found no difference in Forgotten Joint Score (FJS) at 12 months (adjusted mean difference −1.5; 95% CI −7.5 to 4.5), with higher cost in the robotic arm [148]. The registry-nested RASKAL trial found no difference in the Knee injury and Osteoarthritis Outcome Score (KOOS-12) at 2 years between robotic and computer-assisted surgery [149], and the longest robotic TKA RCT (mean 13 years) found no differences in scores or survivorship [150]. No robotic TKA study has shown PROM differences that reach the minimal clinically important difference (MCID) [151], although a meta-analysis of joint awareness found consistently higher FJS with robotics, of uncertain clinical importance [152]. Registry data show no reduction in 5-year revision with navigated or robotic TKA [142].

### 7.2. Dislocation and Higher-Risk Subgroups

The clearest clinical signal in THA is a stability endpoint rather than a biomaterial one. Revision for dislocation was halved with robotic assistance in the American Joint Replacement Registry (hazard ratio [HR] 0.5) without a difference in all-cause revision, and was reduced with navigation in the Australian registry (HR 0.46) [153,154]. Benefits also concentrate in subgroups, including navigated TKA in patients younger than 65 years [155] and robotic posterior-approach THA performed by early-career surgeons [156]. Because most dislocated hips have cups within the Lewinnek zone, stability depends on spinopelvic mobility, which patient-specific functional targeting can address [62,157].

### 7.3. The Contested Alignment Target

The target itself is contested. A mechanical axis within ±3° did not improve 15- or 20-year survival of cemented TKA [158,159], and kinematic or functional alignment yields at most sub-MCID PROM differences but requires fewer soft-tissue releases [160,161,162]. Robotic assistance combined with personalized alignment improved the Oxford Knee Score, whereas robotic mechanical alignment did not [163,164], so trials executed against mechanical targets may test robotics where its advantage is smallest [148]. From a biomaterials perspective, personalized alignment deliberately accepts joint-line obliquity and tibial component varus that the conventional-polyethylene and cementless-migration literature associates with higher wear and migration [14,37]. Its long-term effect on bearing and interface performance is therefore an open question rather than a settled benefit.

## 8. Unicompartmental Knee Arthroplasty: A Potentially Stronger Use Case

UKA may be the strongest use case for enabling technology because small resection tolerances and historically higher revision rates leave more scope for positioning to matter. In an RCT, robotic assistance increased the proportion of components within 2° of target in all planes (for example, tibial sagittal alignment 80% versus 22%) [165], and comparative series report fewer outliers [166,167,168]. Survivorship data are more encouraging than in TKA. The Australian registry reported lower overall revision (HR 0.58) and lower revision for aseptic loosening (HR 0.34) with robotic UKA, although revision for infection was higher and the advantage narrowed against the best-performing conventional implant [169]. Series report 10-year survivorship of 91.7% to 96.4% [170,171], the only RCT with 5-year follow-up found fewer reinterventions (0% versus 9%) without PROM differences [172], and meta-analyses report lower revision and aseptic loosening [5,173]. A propensity-matched database study, however, found no 2-year difference [174]. Navigation-assisted UKA, in which bone preparation remains manual, showed no revision advantage in UK registry data [175], and the 2022 American Academy of Orthopaedic Surgeons guideline found no difference in outcomes between navigated and conventional knee arthroplasty and no significant short-term difference in function, outcomes, or complications between robotic and conventional UKA [176]. The lower aseptic loosening signal is consistent with the fixation-sensitivity argument above, but it comes from non-randomized comparisons.

## 9. Detecting Clinical Effects: Measurement and Statistical Robustness

Two properties of the evidence make a small, late biomaterial benefit difficult to detect. First, PROMs show ceiling effects after arthroplasty. One year after TKA, 26.8% of patients reached the ceiling of the WOMAC pain score [177], and the pain and activities-of-daily-living subscales of the KOOS showed ceiling effects [178]; after THA, 47.5% reached the ceiling of the EuroQol 5-Dimension 3-Level (EQ-5D-3L) instrument [179], and the Oxford Hip Score also showed ceiling effects [180]. The FJS reduces but does not remove this problem [178,180,181,182], and MCID estimates vary widely with the calculation method [183]. Wearable sensors have detected biomechanical differences after robotic TKA that PROMs did not [184], and daily remote monitoring offers a further objective measure of recovery [185]. Second, many arthroplasty RCT findings are statistically fragile. The median fragility index was 1 across total joint RCTs [186] and 3 for robotic versus conventional TKA RCTs, with loss to follow-up exceeding the index in most outcomes [187]; trials comparing revision after manual versus robotic or navigated THA had a median reverse fragility index of 5 [188]. Simulation suggests that detecting a plausible survivorship difference of 1.4% to 2.0% at 15 years would require 2500 to 4000 patients per arm [189]. The absence of a demonstrated survivorship benefit should therefore not be read as evidence of equivalence, particularly for wear-related failure, which emerges beyond the follow-up of current trials.

## 10. Practical Considerations: Learning Curve, Complications, and Cost

Adoption carries costs that must be weighed against uncertain long-term benefits. The learning curve for operative time ranges from roughly 6 to 61 cases, whereas alignment accuracy is maintained from the first case [190,191]. CT-based planning adds radiation exposure and cost that imageless systems avoid [54,192]. Pin-related complications occur in about 1% of cases and are reduced by intra-incisional or unicortical periarticular placement [193,194,195,196]; device-specific adverse events include unexpected robotic-arm movement [197], and periprosthetic fracture signals after robotic THA are conflicting [198,199]. Cost-effectiveness depends strongly on volume and becomes favorable above roughly 24 to 49 cases per year in modeling [200,201]. An English threshold analysis concluded that robotic systems must improve PROMs—not just reduce revision—to be cost-effective [202], whereas US data show lower episode spending at high volume and possible savings in THA [203,204,205,206,207]. The National Institute for Health and Care Excellence recommends only conditional adoption during a three-year evidence-generation period [208].

## 11. Evidence Gaps and Research Priorities

The pivotal unanswered question is whether technology-assisted positioning reduces wear-, corrosion-, or loosening-related revision over the 15- to 20-year horizon at which these failures appear. Two small randomized RSA trials of cemented TKA found no reduction in tibial migration with navigation or robotics [48,49]; no comparable data exist for cementless components, and wear has not been measured directly in matched robotic and conventional cohorts with adequate follow-up [189]. The framework in Figure 1 suggests five priorities. First, randomized comparisons of robotic and conventional implantation should use RSA migration as the primary endpoint with prespecified, validated thresholds [11,12,141], particularly for cementless, HA-coated, and additively manufactured components. Second, preclinical testing should be clinically representative: simulator protocols should reproduce the distribution of positions achieved with manual and robotic techniques, including combined rotational and translational malposition, and should assess damage modes such as rim cracking alongside gravimetric wear [69,70,71]. Third, retrieval analyses should link CT-measured in vivo position to damage modes in HXLPE, vitamin E-stabilized polyethylene, and ceramic bearings. Fourth, registries should record implantation technique together with bearing material, fixation, and coating so that technology effects can be estimated within position-sensitive subgroups rather than across all implants [39,142]. Fifth, trials of personalized alignment should include wear and migration endpoints, and objective functional measures should complement ceiling-limited PROMs [162,163,164,184,185].

## 12. Limitations

This review has limitations. It is a narrative synthesis without quantitative pooling or formal risk-of-bias assessment. Although the search and evidence hierarchy are documented, only PubMed/MEDLINE was searched systematically, records were not screened exhaustively or in duplicate, and study selection and weighting involve judgment, so relevant studies may have been missed and selection bias is possible. Much of the mechanistic evidence comes from simulator, finite-element, animal, and retrieval studies, whose conditions differ from each other and from contemporary robotic practice. Few studies stratify biomaterial outcomes by implantation technique, so most links in the conceptual model are inferred from separate bodies of literature. Finally, the technology is changing rapidly, and platform-specific results may not generalize across systems [3].

## 13. Conclusions

Navigation-assisted and robotic systems consistently improve radiographic positioning accuracy and reduce outliers in THA, TKA, and UKA [2,61,165]. Malposition and edge loading are associated with accelerated wear or metal release for conventional polyethylene, CoC, and MoM bearings in retrieval, cohort, and simulator studies [13,27,31], and varus alignment is associated with greater early migration of cementless tibial components, a validated predictor of loosening [11,37]. However, randomized RSA trials in cemented TKA have not shown less migration with navigation or robotics [48,49]. It has not been established that these technical gains reduce wear-related or aseptic revision with contemporary materials. At mid-term follow-up, randomized and registry data show no consistent reduction in all-cause revision or clinically important improvement in PROMs after TKA or THA. The exceptions are lower dislocation rates after THA, a kinematic rather than biomaterial endpoint, and a more favorable but largely non-randomized survivorship signal in UKA [148,153,169].

We propose two explanations, both requiring confirmation. Highly cross-linked polyethylene appears to have attenuated the wear penalty of modest malposition, although rim fatigue and impingement remain position-dependent [18,21,92]. In addition, current trials are underpowered for late failure [187,189]. On this basis, positioning precision is most likely to matter where the material or interface remains position-sensitive: ceramic-on-ceramic bearings exposed to translational mismatch, cementless and HA-coated fixation, and personalized alignment targets that deliberately depart from neutral. Testing this will require position-stratified RSA and retrieval studies, clinically representative simulator protocols, and registries that link implantation technique to bearing material and fixation methods.

## Figures and Tables

**Table 1:** Evidence tiers used to classify the cited literature.

Tier	Study Designs	What the Tier Can Establish	Principal Limitations for This Question
I	RCTs; meta-analyses of RCTs; national arthroplasty registries	Effect of technology on PROMs, revision, and dislocation; clinical effect of materials	Underpowered for late wear-related failure; mid-term follow-up; registry confounding and limited position data
II	RSA studies; prospective cohorts measuring radiographic wear or metal ions	In vivo wear and early migration; migration thresholds validated against revision	Surrogate endpoints; small samples; rarely stratified by implantation technique
III	Retrieval analyses; retrospective clinical series	In vivo damage modes; association of measured position with wear or failure	Selection of failed implants; position often measured late; confounding
IV	Hip and knee simulators; FEA; cadaveric and animal studies	Mechanism and dose–response under controlled conditions	Standardized loading may not reproduce in vivo kinematics; variable model validation; animal-to-human extrapolation

FEA, finite-element analysis; PROM, patient-reported outcome measure; RCT, randomized controlled trial; RSA, radiostereometric analysis.

**Table 2:** Position sensitivity of bearing materials and fixation interfaces: mechanisms, key position-dependent evidence (with evidence tiers), and clinical translation.

Material or Interface	Position-Sensitive Mechanism	Key Position-Dependent Evidence (Tier)	Clinical Translation
Conventional PE	Wear accelerated by cup abduction, tibial varus, and posterior slope; particle-driven osteolysis	About 40% more linear wear with cup abduction >45° [13]; about twofold penetration with tibial varus >3° [14] (III); +145.9% peak contact stress with 5° varus tilt [15] (IV)	Association clinically established; material largely superseded
HXLPE	Wear largely orientation-insensitive; rim fatigue cracking and impingement damage persist (oxidation of annealed liners is time- and processing-dependent)	No orientation–wear correlation at 10–12 years [16,17] (II); rim cracking with vertical cups [18,19] (III); reduced fatigue crack resistance [20] (IV)	Wear-related revision markedly reduced [21,22] (I); residual risk of rim failure in steep or thin liners
Vitamin E-stabilized PE	As for HXLPE, with greater oxidative stability	No wear change at 80° inclination in a simulator, equally absent for conventional PE [23] (IV); higher wear with anteversion ≥25° [24] (II)	No consistent clinical advantage over HXLPE to date [25,26] (I)
CoC	Microseparation and edge loading cause stripe wear, squeaking, and fracture	Alumina wear increased more than tenfold with microseparation [27] (IV); stripe wear more dependent on anteversion than inclination [28] (III); squeaking inconsistently linked to position [29,30] (III)	Rare but position-linked failures; translational factors (offset, center of rotation) matter
MoM	Edge loading increases metal ion release and adverse reactions to metal debris	Higher ion levels with inclination >55° and insufficient version [31,32] (II–III); 17-fold simulator wear with steep cup plus microseparation [33] (IV)	Clearest clinical position–degradation link; bearing now rarely used
Head–neck taper (metal-on-PE)	Mechanically assisted crevice corrosion	Predicted by alloy combination, neck rigidity, and time [34] (III); no association with cup inclination [35] (II)	Position not an established driver
Cementless porous and 3D-printed surfaces	Micromotion beyond the ingrowth window; varus loading; under-seating or undersizing	+79% peak micromotion with 2° tibial varus [36] (IV); greater migration of varus uncemented tibiae [37,38] (I–II)	No 2-year revision difference between robotic and manual cementless TKA in a registry [39] (I)
HA coating	Micromotion and shear may accelerate resorption and delamination; third-body particles	Thickness and crystallinity govern strength and coating loss [40,41] (IV); HA particles in retrieved PE [42] (III)	Reduced tibial migration [43] (I); higher aseptic cup revision in one registry [44] (I); interaction with position untested
Cemented tibia	Varus overload of proximal tibial bone; cement–implant debonding	Varus >3° associated with medial collapse [45] (III); failure driven by bone fatigue [46] (IV); debonding in well-aligned knees [47] (III); no migration benefit with navigation or robotics in RSA trials [48,49] (I)	Neutral alignment associated with lower failure [50,51] (III); debonding not prevented by alignment

Roman numerals indicate evidence tiers (Table 1). CoC, ceramic-on-ceramic; HA, hydroxyapatite; HXLPE, highly cross-linked polyethylene; MoM, metal-on-metal; PE, polyethylene; TKA, total knee arthroplasty.

## Data Availability

No new data were created or analyzed in this study. Data sharing is not applicable to this article.

## Abbreviations

The following abbreviations are used in this manuscript:

BMI	Body mass index
CI	Confidence interval
CoC	Ceramic-on-ceramic
CT	Computed tomography
EQ-5D-3L	EuroQol 5-Dimension 3-Level
FEA	Finite-element analysis
FJS	Forgotten Joint Score
HA	Hydroxyapatite
HR	Hazard ratio
HXLPE	Highly cross-linked polyethylene
ISO	International Organization for Standardization
KOOS	Knee injury and Osteoarthritis Outcome Score
MCID	Minimal clinically important difference
MoM	Metal-on-metal
PE	Polyethylene
PROM	Patient-reported outcome measure
PSI	Patient-specific instrumentation
RCT	Randomized controlled trial
RR	Risk ratio
RSA	Radiostereometric analysis
THA	Total hip arthroplasty
TKA	Total knee arthroplasty
UKA	Unicompartmental knee arthroplasty

## Appendix A

Table A1 lists the PubMed/MEDLINE searches used to identify and check the literature (Section 2.1).

**Table A1:** PubMed/MEDLINE searches, from inception to 28 September 2026.

Search	PubMed Query	Records
1. Technology, procedure, outcome	(robot*[tiab] OR navigat*[tiab] OR “computer-assisted”[tiab]) AND (“hip arthroplasty”[tiab] OR “knee arthroplasty”[tiab] OR “hip replacement”[tiab] OR “knee replacement”[tiab] OR unicompartmental[tiab]) AND (alignment[tiab] OR position*[tiab] OR wear[tiab] OR fixation[tiab] OR migration[tiab] OR revision[tiab] OR survivorship[tiab] OR outcome*[tiab]) AND english[la]	4151
2. Malposition and degradation	(“hip arthroplasty”[tiab] OR “knee arthroplasty”[tiab] OR “hip replacement”[tiab] OR “knee replacement”[tiab] OR unicompartmental[tiab]) AND (malalign*[tiab] OR malposition*[tiab] OR “component position*”[tiab] OR “edge loading”[tiab] OR inclination[tiab] OR impingement[tiab]) AND (wear[tiab] OR corrosion[tiab] OR loosening[tiab] OR migration[tiab] OR micromotion[tiab] OR retrieval[tiab]) AND english[la]	1222
3. Materials and study design	(“hip arthroplasty”[tiab] OR “knee arthroplasty”[tiab] OR “hip replacement”[tiab] OR “knee replacement”[tiab] OR unicompartmental[tiab]) AND (polyethylene[tiab] OR “cross-linked”[tiab] OR “vitamin E”[tiab] OR ceramic[tiab] OR hydroxyapatite[tiab] OR porous[tiab] OR tantalum[tiab] OR tribocorrosion[tiab]) AND (simulator[tiab] OR retrieval[tiab] OR radiostereometr*[tiab] OR “finite element”[tiab] OR registry[tiab] OR randomized[tiab]) AND english[la]	1488

[tiab], title/abstract field; [la], language. Searches were run on 28 September 2026. Reference lists of included reviews and key trials were hand-searched, and targeted searches were performed for registry reports and guidelines.
